# Mapping the Protein Phosphatase 1 Interactome in Human Cytomegalovirus Infection

**DOI:** 10.3390/v16121961

**Published:** 2024-12-21

**Authors:** Stefan Weinberger, Carmen Stecher, Marie-Theres Kastner, Sergei Nekhai, Christoph Steininger

**Affiliations:** 1 Division of Infectious Diseases and Tropical Medicine, Department of Medicine I, Medical University of Vienna, 1090 Vienna, Austria; 2 Center for Sickle Cell Disease, Howard University, Washington, DC 20059, USA; 3 Karl-Landsteiner Institute of Microbiome Research, 3100 St. Pölten, Austria

**Keywords:** cytomegalovirus, protein phosphatase 1, interactome, proteomics, intracellular trafficking

## Abstract

Protein phosphorylation is a crucial regulatory mechanism in cellular homeostasis. The human cytomegalovirus (HCMV) incorporates protein phosphatase 1 (PP1) into its tegument, yet the biological relevance and mechanisms of this incorporation remain unclear. Our study offers the first characterization of the PP1 interactome during HCMV infection and its alterations. Using co-immunoprecipitation, mass spectrometry, and quantitative proteomics, we identified 159 high-confidence interacting proteins (HCIPs) in the PP1 interactome, consisting of 126 human and 33 viral proteins. We observed significant temporal changes in the PP1 interactome following HCMV infection, including the altered interactions of PP1 regulatory subunits. Further analysis highlighted the central roles of these PP1 interacting proteins in intracellular trafficking, with particular emphasis on the trafficking protein particle complex and Rab GTPases, which are crucial for the virus’s manipulation of host cellular processes in virion assembly and egress. Additionally, our study on the noncatalytic PP1 inhibitor 1E7-03 revealed a decrease in PP1’s interaction with key HCMV proteins, supporting its potential as an antiviral agent. Our findings suggest that PP1 docking motifs are critical in viral–host interactions and offer new insights for antiviral strategies.

## 1. Introduction

Protein phosphorylation is a pivotal regulatory mechanism in eukaryotic cells, affecting over 70% of cellular proteins. This dynamic process is tightly controlled by the opposing actions of kinases and phosphatases. Dephosphorylation is predominantly executed by a limited number of protein serine/threonine phosphatases (PP1-7), vital for regulating the phosphorylation status of numerous phosphoproteins. Among these, the highly conserved and abundant Ser/Thr protein phosphatase 1 (PP1) plays a major role, responsible for dephosphorylating about one-third of all eukaryotic proteins [1]. PP1 operates as a holoenzyme, comprising a catalytic subunit, PP1c, along with various regulatory subunits (PPP1R). This assembly is integral for dictating PP1’s specific roles in cellular processes. Despite significant homology among PP1c isoforms, the precise spatial and temporal targeting of PP1 to distinct substrates is largely determined by its interaction with respective regulatory subunits [1,2]. PP1 engages with these subunits primarily through conserved docking motifs, the most common of which are RVxF, SILK, and MyPhoNE, among others [3]. These interactions are crucial in aligning the PP1 catalytic subunit with the PPP1R subunit and modulating PP1’s activity and substrate specificity [1].

Various viruses have developed strategies to exploit these regulatory processes. They achieve this either by expressing viral PPP1Rs or inducing the synthesis of host PPP1Rs, thereby creating conditions favorable for their replication [4,5]. For instance, the measles virus hinders antiviral type I interferon production by targeting PP1, limiting the dephosphorylation and activation of RIG-I-like receptors and MDA5 [6]. The respiratory syncytial virus (RSV) subverts PP1 to regulate viral transcription through the RSV P protein, which interacts with PP1 via an RVxF-like motif [7]. Similarly, herpes Simplex Virus 1 produces ICP34.5 to augment eIF2α dephosphorylation [8], while African swine fever virus synthesizes DPL71 protein to serve a similar function [5].

Among these viral manipulations, HCMV, a member of the herpesvirus family, engages in a unique interaction with PP1. Notably, HCMV incorporates host PP1 into the mature viral particles’ tegument layer, triggering cellular hypophosphorylation upon viral entry [9,10]. The broader biological relevance and implications of this HCMV-associated activity remain to be understood. In immunocompetent individuals, HCMV typically remains dormant, but it can cause life-threatening diseases in immunocompromised patients, such as those with HIV/AIDS or transplant recipients. Moreover, congenital HCMV infection is a leading cause of congenital disability in newborns. Given that HCMV’s high seroprevalence reaches up to 90% in some geographic regions [11], as well as its propensity for reactivation, it is crucial to understand the viral interactions with host proteins such as PP1. Targeting critical virus–host protein interactions with small molecules, such as the interactions between antiviral restriction factors (ARFs) and viral antagonists, can significantly enhance endogenous infection control [12]. We recently demonstrated that 1E7-03, a noncatalytic inhibitor of PP1 known to bind to the RVxF-accommodating PP1 cavity, effectively inhibits HCMV replication in vitro [13]. Importantly, this inhibitor exhibited a nontoxic profile during infection, contrasting with other catalytic PP1 inhibitors and underscoring its potential in antiviral treatment [14].

Thus, a comprehensive analysis of the PP1 interactome involving host and viral proteins holds significant potential for antiviral treatment strategies, particularly for HCMV, where existing antiviral treatment options are limited. In this study, utilizing co-immunoprecipitation coupled with label-free quantification (LFQ) mass spectrometry analysis and bioinformatics approaches, we characterized the PP1 interactome during HCMV infection and its temporal alterations. Our aim was to elucidate the intricate interplay between viral and host proteins and to understand how HCMV may leverage PP1 to facilitate its replication. Additionally, we examined the effects of the noncatalytic PP1 inhibitor 1E7-03 on the PP1 interactome during HCMV infection, shedding light on the mechanisms underlying its antiviral action.

## 2. Materials and Methods

### 2.1. HCMV Infection

Human cytomegalovirus (HCMV, strain AD169) was prepared according to previously established protocols [15]. Human foreskin fibroblasts (HFFs) were cultured in 175 cm^2^ tissue culture flasks using Dulbecco’s Modified Eagle Medium (DMEM GlutaMAX, Thermo Fisher Scientific, Waltham, MA, USA) supplemented with 10% fetal calf serum (FCS) and Antibiotic-Antimycotic mix (Thermo Fisher Scientific). For the PP1 inhibition studies, HFF cells were pretreated with 1E7-03 20 μM for one hour prior to infection. Subsequently, both vehicle-treated (DMSO) and 1E7-03-pretreated HFF cells were infected in triplicate using the HCMV laboratory strain AD169, with a multiplicity of infection (MOI) set at 3 to ensure a significant infection rate. One hour post-infection, the viral suspension was replaced with culture medium.

### 2.2. Co-Immunoprecipitation

Cells were harvested at two time points, 4 h and 96 h, post-infection. Infected cells were washed with phosphate-buffered saline (Thermo Fisher Scientific) and harvested with a cell scraper. For the co-immunoprecipitation (Co-IP) of PP1, HFF cells were lysed in a buffer containing 20 mM Tris-HCl pH 7.4, 137 mM NaCl, 1% NP-40 supplemented with 1% Halt protease, and phosphatase inhibitor (Thermo Fisher Scientific), and then rotated for 30 min at 4 °C. Additionally, nuclear lysis was facilitated through sonication pulses utilizing a Bioruptor Plus bath sonicator (Diagenode, Denville, NJ, USA) for 30 s. Cellular debris was pelleted by centrifugation at 10.000× *g* for 10 min at 4 °C. Subsequently, after taking aliquots for input samples, protein concentrations were determined using the Pierce BCA assay (Thermo Fisher Scientific). Co-IP was performed by adding 1 μg of the respective antibody, PP1 Antibody (E-9) #sc-7482, and control IgG (both from Santa Cruz Biotechnology, Dallas, TX, USA), for 1 h at 4 °C, followed by the addition of Protein A/G PLUS-Agarose beads (Santa Cruz Biotechnology) for overnight incubation at 4 °C. The beads were then washed five times with phosphate-buffered saline (Thermo Fisher Scientific).

### 2.3. SDS-PAGE and Western Blot

Beads were subjected to a 5 min incubation at 90 °C in Laemmli sample buffer supplemented with β-mercaptoethanol. Equal amounts of sample were loaded onto 4–15% precast Mini Protean TGX polyacrylamide gradient gels (Bio-Rad, Hercules, CA, USA) and subjected to SDS-PAGE. Gels were run at a constant 100 V for 80 min. Proteins were then blotted onto a 0.45 μm PVDF membrane at 100 V for 1 h (Thermo Fisher Scientific) using a wet tank with Tris/Glycine buffer containing 20% methanol. Membranes were stained with Ponceau S solution to confirm the blotting efficiency and to visualize the total protein amount. Destained membranes were treated with StartingBlock TBS blocking buffer (Thermo Fisher Scientific), and incubated overnight with primary antibodies at 4 °C. Following incubation with HRP-linked secondary antibodies and subsequent rinses in 1 × TBS, the blots were visualized using a ChemiDoc (Bio-Rad) with SuperSignal Pico substrates (Thermo Fisher Scientific). The monoclonal antibody PP1 (E-9) #sc-7482 was procured from Santa Cruz Biotechnology. Additionally, the monoclonal antibody targeting PPP1R7 (clone OTI4F9) was sourced from Invitrogen (Waltham, MA, USA), and the polyclonal antibody for TRAPPC3 (catalog #15555-1AP) was acquired from Proteintech (Rosemont, IL, USA). For immunodetection, the secondary antibodies included goat anti-rabbit HRP (catalog #7074) from Cell Signaling Technology (Danvers, MA, USA) and goat anti-mouse HRP (catalog #1706516) from Bio-Rad.

### 2.4. Sample Preparation for Mass Spectrometry Analysis

After transferring the beads to a 0.2 mL PCR tube and removing the buffer, the beads were resuspended in 30 µL 2 M urea and 50 mM ammonium bicarbonate. Proteins were digested with 75 ng of LysC (mass spectrometry grade, FUJIFILM Wako chemicals, Richmond, VA, USA) and 75 ng of trypsin (Trypsin Gold, Promega, Madison, WI, USA) at room temperature in the dark for 90 min. The supernatant was then transferred to a new 0.2 mL PCR tube, and the beads were rinsed once with 30 µL 1 M urea and 50 mM ammonium bicarbonate. Disulfide bonds were reduced with 2.4 µL of 250 mM dithiothreitol (DTT) for 30 min at room temperature, followed by alkylation with 2.4 µL of 500 mM iodoacetamide for 30 min at room temperature in the dark. The excess iodoacetamide was quenched with 1.2 µL of 250 mM DTT for 10 min. After adding 30 µL of 50 mM ammonium bicarbonate, the proteins were digested overnight at 37 °C with 75 ng of LysC and 75 ng of trypsin. The digestion was stopped by the addition of trifluoroacetic acid (TFA) to a final concentration of 0.5%, and the peptides were desalted using C18 Stagetips [16].

### 2.5. Liquid Chromatography–Mass Spectrometry Analysis

Peptides were separated on an Ultimate 3000 RSLC nano-flow chromatography system (Thermo Fisher Scientific), employing a pre-column for sample loading (Acclaim PepMap C18, 2 cm × 0.1 mm, 5 μm, Thermo Fisher Scientific), and a C18 analytical column (Acclaim PepMap C18, 50 cm × 0.75 mm, 2 μm, Thermo Fisher Scientific). A segmented linear gradient of 2% to 35%, and finally to 80% solvent B (80% acetonitrile, 0.1% formic acid; solvent A: 0.1% formic acid) was applied at a flow rate of 230 nL/min over 120 min. The eluted peptides were analyzed on an Exploris 480 Orbitrap mass spectrometer (Thermo Fisher Scientific) coupled to the column with a FAIMS pro ion source (Thermo Fisher Scientific) using coated emitter tips (PepSep, MSWil, Aarle-Rixtel, The Netherlands) with the following settings: The mass spectrometer operated in data-dependent acquisition (DDA) mode with two field asymmetric ion obility Spectrometry (FAIMS) compensation voltages (CV) set to −45 or −60 and a 1.5 s cycle time per CV. Survey scans were obtained in a mass range of *m*/*z* 350–1500, at a resolution of 60k at *m*/*z* 200, with a normalized Automatic Gain Control (AGC) target at 100%. The most intense ions were selected with an isolation width of *m*/*z* 1.2, fragmented in the HCD cell at 28% collision energy, and spectra were recorded for max. 100 ms at a normalized AGC target of 100% and a resolution of 15k. Peptides with a charge of +2 to +6 were included for fragmentation. The peptide match feature was set to ’preferred’, the exclude isotope feature was enabled, and selected precursors were dynamically excluded from repeated sampling for 45 s.

### 2.6. Bioinformatic Data Analysis of Mass Spectrometry Data

MS raw data split for each CV using FreeStyle 1.8 SP2 (Thermo Fisher Scientific) were analyzed using the MaxQuant software package version 2.1.4.0 [17] against the Uniprot human reference proteome (version 2022_04, accessed on 8 September 2023, www.uniprot.org) and the Uniprot Human herpesvirus 5 reference proteome (version 2022_04), alongside a database of most common contaminants. The search was performed with full trypsin specificity and a maximum of two missed cleavages at a protein and peptide spectrum match false discovery rate of 1%. Carbamidomethylation of cysteine residues was set as a fixed modification, while oxidation of methionine, and N-terminal acetylation were considered variable modifications. Label-free quantification (LFQ) was performed with the “match between runs” feature and the LFQ function enabled, while other parameters remained at their default settings. MaxQuant output tables were further processed in RStudio (https://posit.co/products/open-source/rstudio/) using R 4.2.2 (https://www.R-project.org) and the Cassiopeia_LFQ script (https://github.com/maxperutzlabs-ms/Cassiopeia_LFQ), all accessed on 8 September 2023. Data cleaning involved the removal of reverse database identifications, contaminant proteins, protein groups identified only by modified peptides, protein groups with fewer than two quantitative values in any experimental group, and protein groups with less than two razor peptides. Missing values were imputed by randomly drawing data points from a normal distribution model on the whole dataset (data mean shifted by −1.8 standard deviations, with a distribution width of 0.3 standard deviations). Statistical differences between groups were evaluated using the LIMMA 3.54.0 [18], applying a batch correction at 5% FDR (Benjamini-Hochberg). Further data exploration and visualization were carried out using the amica proteomics data analysis platform [19].

### 2.7. Functional Proteomic Data Analysis

The heatmap was generated using the seaborn library in Python. For the PP1 HCIP motif analysis and validation, three FASTA files, representing 83 PP1-regulatory subunits with the RVxF motif, 7 with the MyPhoNE motif, and 7 with the SILK motif, were processed using MEME Version 5.5.3 [20] with motif-specific parameters. For the RVxF motif, motif occurrence distribution was set at zero or one per sequence, motif width was set between 4 and 5, and a maximum of 5 motifs were to be identified, restricted to the given strand only. For the MyPhoNE and SILK motifs, parameters were similarly adjusted. Subsequently, position weight matrix (PWM) created by MEME were submitted to FIMO (version 5.5.3) [21] for a search against the Co-IP/MS experimental data, considering a *p*-value less than 0.001 as significant. The PWMs obtained using MEME were then loaded into R 4.2.2. Utilizing the ggseqlogo package, sequence logos were generated to graphically illustrate the information content and the conservation level of amino acids at each respective position within the motifs identified from the analysis. The protein data was organized in three groups, each representing one of the PP1 binding motifs: RVxF, MyPhoNE, and SILK. Venn diagrams were generated using the eulerr R package (accessed 20 December 2023, https://github.com/jolars/eulerr) [22].

## 3. Results

### 3.1. Identification of PP1 Interactors

**Figure 1:**
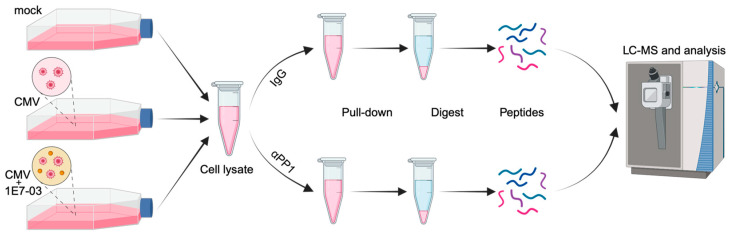
The PP1 interactome was analyzed through co-immunoprecipitation, liquid chromatography-mass spectrometry, quantitative proteomics and bioinformatics. Samples were collected at 4 h and 96 h post-infection (p.i.), in triplicate, under three conditions: mock-infected HFF, HCMV AD169-infected HFF, and HFF infected post 1E7-03 pretreatment.

To investigate the PP1 interactome and PP1 regulatory subunits during HCMV infection, we adapted an unbiased approach for profiling PP1 interactors based on label-free quantification [23] (Figure 1). This protocol was first refined using lysates from uninfected HFF cells, and then applied to samples from HCMV-infected HFF cells at selected time points. Analysis was performed at the 4 h and 96 h time points post-infection to capture both immediate early interactions with host mechanism and extensive host cell reprogramming and viral replication dynamics.

**Figure 2:**
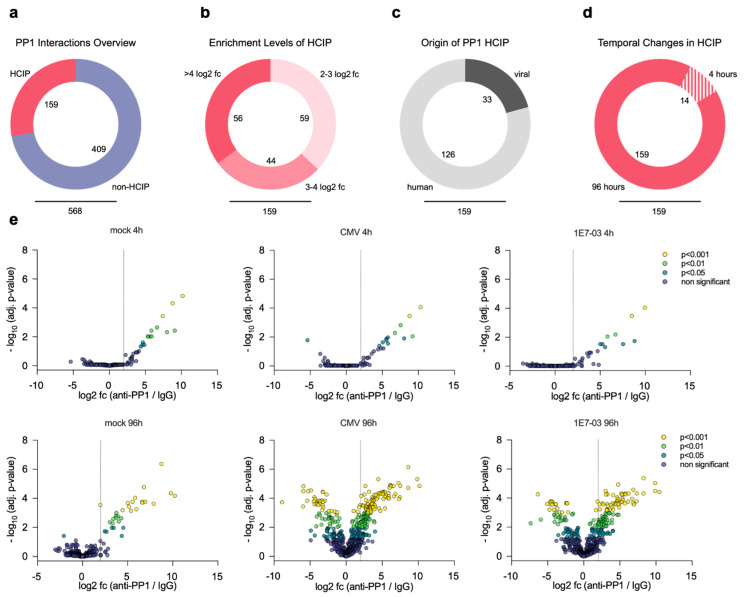
(**a**) Total number of proteins and HCIP identified by mass spectrometry (**b**) Proportions of HCIP enrichment levels (**c**) Abundance proportions of host and viral PP1 HCIP. (**d**) Temporal distribution of HCIP, showing early (4 h) and late (96 h) interactors. (**e**) Mock infected HFF or HCMV infected HFF or PP1 inhibitor pretreated HCMV infected HFF were lysed and immunoprecipitated with anti-PP1 versus isotype-control IgG 4 h p.i. or 96 h p.i. and analyzed by MS. The *x*-axis plots the log2 fold change in anti-PP1 to IgG control. The *y*-axis shows the −log10 of the adjusted *p*-values. Proteins with a ratio > 2 and an adjusted *p*-value < 0.05 are considered PP1 interactors.

In total, we identified and quantified 568 proteins from lysates of HFF and cytomegalovirus infected HFF after Co-IP. Notably, 159 of these proteins were at least 2-fold enriched in PP1 pulldown assays and showed significant changes compared to the IgG control pulldowns, indicating specific binding to PP1 (Figure 2a,b and Table S1 in Supplementary Data). Among these PP1 high-confidence interacting proteins (HCIP), 126 are of human origin and 33 are viral proteins (Figure 2c).

**Figure 3:**
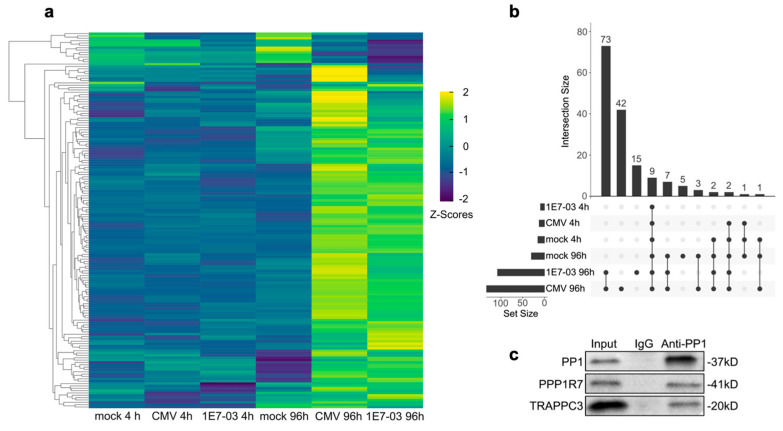
(**a**) Heatmap displays mean z-scores of 159 HCIP, each at least 2-fold enriched (adjusted *p*-value < 0.05). Z-scores, calculated from triplicate measurements, are color-coded from −2 (blue) to 2 (yellow) as shown in the color bar. Proteins are ordered based on hierarchical clustering. (**b**) UpSet plot visualizing overlap of significant proteins from different conditions. An isolated dot denotes the number of proteins unique to that particular group. The bar chart at the top indicates the count of proteins that intersect among sets, while the sidebar chart represents the quantity of differentially abundant proteins in each comparison. (**c**) Co-immunoprecipitation and subsequent Western blot analyses from lysates of HCMV infected HFF with two PP1 HCIP, PPP1R7 and TRAPPC3.

Initially, at the immediate early stages of infection (4 h), the differences in PP1–protein interactions between conditions were not markedly distinct (Figure 2d,e), suggesting a consistent trend in PP1–protein interactions regardless of infection or treatment status. However, at the late-stage phase of infection (96 h), we observed a significant divergence in protein enrichment profiles, particularly when compared to the 4 h p.i. data. The prolonged duration of infection manifested in significantly altered protein–PP1 interactions, with evident shifts in the enrichment levels which were not seen in the mock 96 h group (Figure 3a). Additionally, the variations observed between the groups undergoing HCMV infection and those receiving inhibitor pretreatment prior to HCMV infection revealed subtle differences with few altered protein–PP1 interactions. Subsequent analysis further delineated these patterns, identifying the largest intersecting subset of 73 HCIP between the HCMV 96 h and 1E7-03 96 h condition groups. Moreover, the HCMV 96 h dataset harbored the highest number (42) of unique HCIP (Figure 3b). In addition to mass spectrometry analysis, we further validated our MS data by co-immunoprecipitation followed by Western blotting. Using lysates from HFF cells, we confirmed specific interactions between PP1 and the known PP1 regulatory subunit PPP1R7 (SDS22), as well as the newly identified PP1 interactor TRAPPC3 (Figure 3c).

### 3.2. Pathway Enrichment of PP1 Interaction Partners

**Figure 4:**
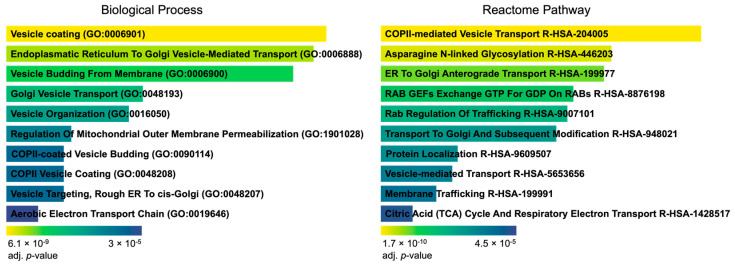
Enrichment analysis of human PP1 HCIP using Enrichr [24] sorted by adjusted *p*-value ranking showing the top 10 hits for biological processes and reactome pathways.

Pathway enrichment analysis of human PP1 HCIP revealed significant alterations in intracellular protein trafficking processes following HCMV infection. These enrichments of PP1 HCIP were particularly evident in processes such as vesicle coating, targeting, and budding (Figure 4). Most notably, pathways mediating transport between the rough endoplasmic reticulum and the cis-Golgi apparatus were prominently affected.

### 3.3. Bioinformatic Analysis of PP1 Binding Partners

**Figure 5:**
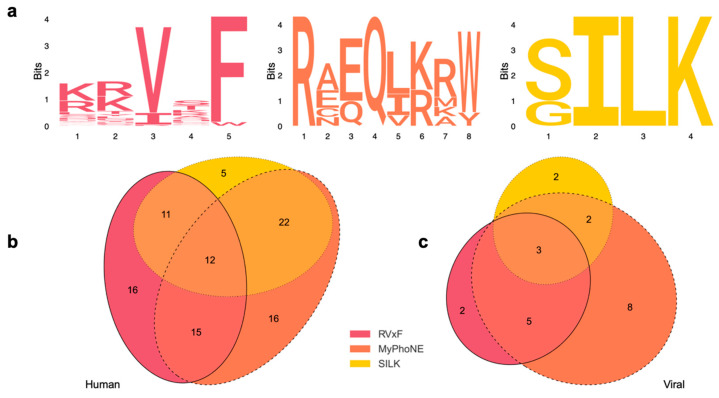
(**a**) Sequence logos of PP1-docking motifs. The logos visualize amino acid conservation among the HCIP across three primary docking motifs of PP1. The *x*-axis denotes residue positions, while the *y*-axis, in bits, reflects the conservation level. Larger letters indicate higher frequency amino acids, emphasizing conserved regions within the motifs. (**b**,**c**) Venn diagrams representing the overlap among human and viral PP1 HCIP that possess the three respective PP1-docking motifs: RVxF, MyPhoNE, and SILK.

Subsequent analysis focused on the 159 HCIP identified, examining their alignment with the following three most prominent PP1-docking motifs: [KR][KR][VI]x[FW] (RVxF), RxxQ[VIL][KR]x[YW] (MyPhoNE), and [GS]IL[RK] (SILK) [25]. Among these 159 HCIP, 120 were found to possess at least one of these docking motifs. Specifically, 22 of these proteins were of HCMV origin and 98 were human proteins (Figure 5a). Notably, a significant proportion of these proteins harbor multiple PP1 docking motifs, with a distinct subset exhibiting all three motifs (Figure 5b,c).

### 3.4. HCMV Alters Interactions of PP1 Regulatory Subunits

**Figure 6:**
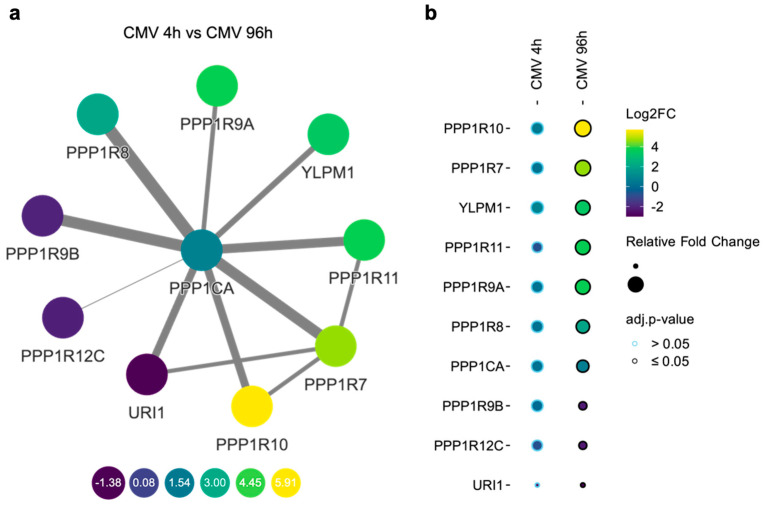
(**a**) Differentially abundant PPP1Rs displayed in a PPI-Network from the IntAct database [26]. Log2 fold changes are displayed as a color gradient of the nodes. Greater edge widths signify higher interaction confidence, as denoted by the Mutual Information score. (**b**) Dotplot indicating log2 fold changes (mapped as color gradient), relative abundance (cir-cle size) and adjusted *p*-values (border color) for all detected PPP1Rs comparing HCMV infection after 4 h vs. HCMV infection after 96 h.

Of the 159 PP1 HCIP, 11 were previously described as PP1 regulatory subunits (PP1Rs), each bearing a PPP1R designation according to the HUGO Gene Nomenclature Committee database. In the HCMV group, all detected PP1Rs exhibited significant temporal differences in the PP1 interactions between the early (4 h) and late (96 h) stages of infection (Figure 6a,b). Protein interaction network mapping revealed dynamic changes in these interactions (Figure 6a). Specifically, the interaction with PPP1R10 (PNUTS) was found to be most enriched over the course of the infection, whereas the interaction with URI1 (PPP1R19) showed the most pronounced decrease.

### 3.5. HCMV Modulates PP1 Interactions in Protein Trafficking

**Figure 7:**
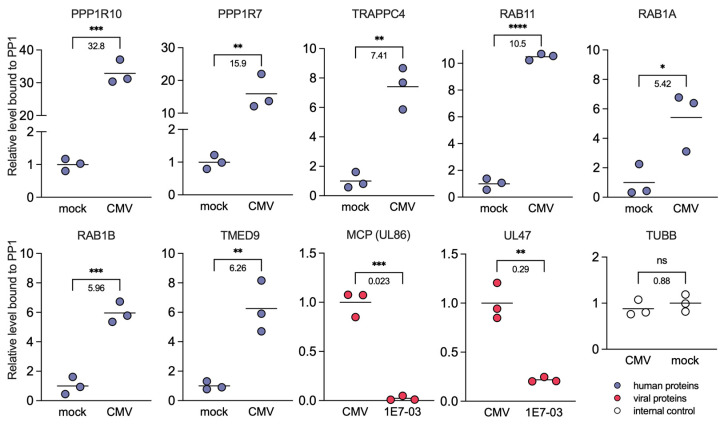
Comparison of binding between PP1 and HCIP during HCMV infection. Alterations in the binding of PP1 interactors 96 h post HCMV infection (*n* = 3). Quantification was derived from LFQ signals, with TUBB serving as an internal control. ns = not significant, * *p* < 0.05, ** *p* < 0.001, *** *p* < 0.0001, **** *p* < 0.00001. Fold change numbers are displayed below the brackets. Comparisons as described on *x*-axis (CMV vs. mock or CMV + 1E7-03 vs. CMV).

We then investigated proteins that had demonstrated the most significant changes in PP1 interaction during HCMV infection. We identified a distinct enrichment of HCIP involved in intracellular protein trafficking processes, specifically TRAPPC4, RAB11, RAB1A, RAB1B, and TMED9 (Figure 7). Intriguingly, pretreatment with 1E7-03 significantly attenuated the interaction of PP1 with key HCMV proteins including the major capsid protein (MCP) UL86 and the inner tegument protein UL47 (Figure 7).

## 4. Discussion

This study presents the first description of the PP1 interactome during an HCMV infection, highlighting its significant alterations. Our objective was to delineate the interactome of protein phosphatase 1 in the context of an HCMV infection, thereby aiming to gain new insights into the biological rationale behind PP1’s incorporation by HCMV. Recent findings have described how PP1 is commandeered to the tegument by UL32, demonstrating that PP1 plays a role in preventing the activation of 14-3-3 proteins [27]. In our study, despite observing some enrichment of UL32, it did not emerge as one of the most abundant viral proteins interacting with PP1. UL32 did not surpass our designated threshold, and thus was not classified as a high-confidence interacting protein of PP1 in our analysis.

We identified 159 proteins, termed as high-confidence interacting proteins, showing at least a 2-fold enrichment in PP1 co-immunoprecipitation during HCMV infection, which significantly expands our current understanding of PP1’s interaction with host cell machinery. Among these, 126 proteins are of human origin, and 33 are viral proteins. This suggests that PP1’s interactions during HCMV infection are broader and more nuanced than previously thought. In the context of our findings, the increasing prevalence of host PP1 interacting partners at a later time point of infection can be attributed to the progressive host cell reprogramming by HCMV. HCMV extensively modulates host cell protein synthesis and cell cycle dynamics to foster an environment conducive to its replication [28]. This viral manipulation, which intensifies over the course of infection, manifests as profound alterations in the host’s transcriptome and proteome [29], leading to a delayed yet extensive PP1 interactome observed predominantly at 96 h p.i. The observed temporal pattern of host protein interactions with PP1 aligns with the progressive viral control over host cell mechanisms, a strategy HCMV employs to ensure its replication and evade host defenses [30]. The distinctive alterations in the PP1 interactome post-infection reflect specific viral–host interactions rather than general shifts in gene expression, ensuring the accuracy of our proteomic findings [17,31,32].

Although we did not observe drastic changes in the PP1 interactome during the early stages of infection, this does not imply an absence of PP1 activity at that phase. Previous studies have shown that PP1 inhibition with 1E7-03 significantly impairs HCMV replication by affecting the metabolic signaling pathways through altered AMP-activated protein kinase dephosphorylation, leading to elongation factor 2 phosphorylation and ultimately reducing viral translation and propagation [13]. Moreover, PP1 has been demonstrated to interact with HCMV immediate early proteins, influencing the PKR/eIF2α signaling axis and promoting efficient viral mRNA translation [33]. Thus, PP1 can exert critical regulatory functions early in infection without necessitating large-scale changes in its interactome.

### 4.1. Known Regulatory Subunits

PP1 catalytic subunits depend on a multitude of regulatory subunits to direct their subcellular localization and determine substrate specificity [2]. Our findings indicate that HCMV infection disrupts PP1 function through the temporal dysregulation of at least nine recognized regulatory subunits, PPP1R10, PPP1R7, YLPM1, PPP1R11, PPP1R9A, PPP1R8 (NIPP1), PPP1R9B, PPP1R12C, and URI1 (Figure 6). Most of these proteins have no documented connection to HCMV, although some are known to play a role in viral infections. In particular, PPP1R12C is noteworthy in the context of viral infections, including those caused by RNA viruses like SARS-CoV-2, influenza, Zika virus, or vesicular stomatitis virus [34]. It has been observed that silencing PPP1R12C leads to enhanced phosphorylation of the pattern recognition receptors RIG-I and MDA5, which are pivotal in the antiviral response, impairing the induction of IFNβ, a crucial antiviral interferon. This suggests that PPP1R12C is necessary for the dephosphorylation and proper functioning of RIG-I-like receptors [34]. Another significant regulatory subunit, NIPP1, also plays a critical role in viral dynamics. In HIV-1 infection, HIV-1 Tat interacts with PP1 through the highly conserved Q^35^VCF^38^ sequence and shuttles PP1 to the nucleus [35]. The ectopic expression of NIPP1, as well as the central domain of NIPP1 (cdNIPP1), as part of HIV-1 genome efficiently inhibited HIV-1 replication [36,37]. Thus, the sequestration of PP1 or targeting it with small molecules to dissociate Tat from PP1 appears to be a feasible approach to inhibit HIV-1 transcription. Indeed, 1E7-03 competed with Tat-derived QVCF sequence containing peptide for binding with PP1 in vitro in a dose-dependent manner and reduced the nuclear targeting of PP1 by Tat in cultured cells thus efficiently blocking HIV-1 transcription [38].

### 4.2. Implications of PP1 Interactions in Viral Trafficking

Our enrichment analysis conducted on all 159 high-confidence interacting proteins of PP1 sheds light on the intricate host–virus interaction landscape, particularly highlighting vesicular trafficking and metabolic modulation as significant aspects of this interaction (Figure 4). Notably, the proteins TRAPPC3, TRAPPC2L, TRAPPC1, TRAPPC6B, TRAPPC2, TRAPPC5, and TRAPPC4 are recurrent across several vesicular trafficking-related biological processes and pathways such as vesicle coating, vesicle budding from membrane, and golgi vesicle transport (Figure 4). These proteins, part of the trafficking protein particle complex, are instrumental in vesicular trafficking and transport between the endoplasmic reticulum and Golgi apparatus [39]. The perturbation of these processes by HCMV underscores the virus’s strategy to hijack host cellular trafficking machinery for its replication and assembly [40,41]. In fibroblasts infected with HCMV, transcriptomic analysis revealed a marked upregulation of cellular transcripts associated with intracellular trafficking mechanisms during the late stages of infection [42,43]. Furthermore, the increased interaction of PP1 and proteins like RAB1A/B and RAB11A/B, which are involved in multiple vesicular trafficking processes and pathways such as COPII-mediated vesicle transport and ER-to-Golgi anterograde transport, highlights the pivotal role of Rab GTPases in vesicular dynamics, which are usurped by HCMV to facilitate its lifecycle [44,45]. Rab proteins are quintessential regulators of membrane trafficking pathways, orchestrating the flow of membranes through the endosomal recycling compartment (ERC) alongside their guanine nucleotide exchange factors (GEFs) and GTPase-activating proteins (GAPs). Rab GTPases have been associated with the replication of various significant viral pathogens in humans [46]. HCMV’s exploitation of Rab cascades to reorganize the ERC and redirect membrane flow exemplifies the virus’s capacity to modulate the host cellular environment for its benefit [47]. The establishment of a virion assembly compartment (VAC) is a hallmark of HCMV infection, and the transition of a cellular membranous system toward this end begins in the early phase of infection, transpiring through to the late phase where the newly formed virions are assembled and released [47]. Observing an enrichment of Rab–PP1 interactions 96 h p.i. suggests a strategic modulation of phosphatase activity by HCMV, potentially impacting key signaling pathways in vesicular trafficking and membrane reorganization, thereby facilitating VAC assembly and egress processes. Another HCIP of PP1 that plays a multifaceted role in the secretory pathway is TMED9 [48]. Suppressing TMED9 simultaneously diminishes autophagic processes and lowers virus production. This effect may be attributed to a mechanism involving COPII-dependent virus transportation [49,50]. These findings highlight how HCMV may co-opt host cellular mechanisms by altering PP1–protein interactions and vesicular trafficking processes, with implications for HCMV assembly and egress.

### 4.3. PP1 Modulation by 1E7-03 in HCMV Infection

The mapping of interactions between PP1 and viral proteins provides an important insight into antiviral research. The inhibitor 1E7-03, specifically targeting the RVxF-interacting site on PP1, has demonstrated a notable impact on the replication of Venezuelan equine encephalitis virus (VEEV), HIV-1, Ebolavirus, Rift Valley fever virus, and HCMV [13,38,51,52,53,54]. Specifically, it was shown that 1E7-03 modulates PP1 interaction with the VEEV capsid protein, affecting its phosphorylation and thus revealing a discernible influence on viral replication dynamics. The treatment with 1E7-03 exhibited a notable reduction in viral titers post-infection, asserting the potential of PP1 RVxF binding pocket as a viable therapeutic target against alphaviruses including VEEV [52]. Recent global proteomic analysis of 1E7-03-treated T cells showed no changes in host protein expression levels [55]. Notably, 1E7-03 affected protein phosphorylation in the PPARα/RXRα canonical pathway which included increased phosphorylation of HLA-B/C, LSM14A, ASPM proteins and decreased phosphorylation of NPM1, p53, HSP90AA1, and HSP90AB1 proteins [55]. Furthermore, 1E7-03 also modulated protein phosphorylation in the TGF-β pathway that included TGF-β2, SMAD7, and PD2 proteins [55]. In our study, the decreased interaction between PP1 and both the HCMV major capsid protein as well as the inner tegument protein UL47 during 1E7-03 treatment is an insightful finding, as it provides evidence that 1E7-03 affects the interaction of PP1 with key viral proteins involved in the assembly process of the virus. While the specific interactions between PP1 and the MCP of HCMV remain largely unexplored, the observed antiviral efficacy of 1E7-03 against HCMV could potentially mirror the mechanism elucidated in VEEV. Reduced binding of PP1 to the HCMV MCP, as induced by 1E7-03, may modulate the phosphorylation landscape of the MCP. This modulation could, in turn, disrupt the precise assembly process of the viral capsid, impeding effective viral replication. Subsequent analyses of PP1 docking motifs within the HCIP elucidated their distribution and conservation. Our study revealed a notable prevalence and conservation of the RVxF, SILK, and MyPhoNE motifs across human and viral HCIP (Figure 5). These findings highlight their potential role in PP1-mediated interactions during HCMV infection. Particularly, the MyPhoNE motif stands out as the most prevalent, suggesting a critical function in these interactions. This characterization advances the understanding of PP1 interactions, serving as a foundation for creating novel PP1 inhibitors and guiding subsequent research on docking motifs to modulate PP1 activity and mitigate viral propagation.

Our investigation highlights significant alterations in the PP1 interactome during HCMV infection. The analysis of phosphorylation status alterations in various proteins is certainly intriguing, yet it extends beyond the current study’s focus. Such analyses represent a promising direction for future studies to build upon the foundational insights we have provided here. While our study provides valuable insights, it is subject to the inherent challenges of mass spectrometry, such as the presence of background proteins and variability among control groups, which may have masked more subtle yet significant alterations in the PP1 interactome. Moreover, co-immunoprecipitation is commonly used for detecting protein–protein interactions, yet it does not differentiate between direct interactions and those mediated via multiprotein complexes. Consequently, results represent associations rather than direct contacts. This limitation is mitigated when assessing the involvement of proteins in biological processes or pathways, where function often arises from protein complex interactions. Additionally, the AD169 strain’s genomic deletions may limit the generalizability of our findings. Future research using a clinical HCMV strain could complement our findings and provide a broader understanding of the PP1 interactome in an HCMV infection.

## 5. Conclusions

In summary, our study identified significant temporal alterations within the PP1 interactome subsequent to an HCMV infection. Given PP1’s critical involvement in a host of cellular functions, these findings pave the way for advancing our understanding of the role of PP1 integration within the HCMV tegument and its biological implications. While the study possesses an exploratory character and refrains from positing a definitive mechanistic explanation for PP1’s incorporation into the HCMV virion, it nonetheless identifies several cellular processes and pathways that are potentially advantageous to HCMV, attributable to the integration of PP1. In particular, cellular trafficking processes emerge as a pivotal area of interest for proteins associated with PP1. Moreover, building upon our previous research that demonstrated the inhibition of HCMV replication via the noncatalytic PP1 inhibitor 1E7-03, we propose an additional mechanistic hypothesis for its antiviral efficacy against HCMV. When integrated with the previous research, the inhibitory effects of small molecule modulators on PP1 emerge as a promising avenue for broad-spectrum antivirals. Our interactome data will be important for future studies of herpesvirus infection.

## Data Availability

The mass spectrometry proteomics data have been deposited to the ProteomeXchange Consortium via the PRIDE partner repository [56] with the dataset identifier PXD046946.

## Supplementary Materials

The following supporting information can be downloaded at: https://www.mdpi.com/article/10.3390/v16121961/s1, Table S1: High-Confidence Interacting Proteins of PP1 Identified by Label-Free Quantification (LFQ) Mass Spectrometry Analysis.

## References

[1] Heroes E., Lesage B., Görnemann J., Beullens M., Van Meervelt L., Bollen M. (2013). The PP1 binding code: A molecular-lego strategy that governs specificity. FEBS J..

[2] Ceulemans H., Bollen M. (2004). Functional Diversity of Protein Phosphatase-1, a Cellular Economizer and Reset Button. Physiol. Rev..

[3] Bollen M., Peti W., Ragusa M.J., Beullens M. (2010). The extended PP1 toolkit: Designed to create specificity. Trends Biochem. Sci..

[4] Bryant K.F., Macari E.R., Malik N., Boyce M., Coen D.M. (2009). ICP34.5-Dependent and -Independent Activities of Salubrinal in Herpes Simplex Virus-1 Infected Cells. Virology.

[5] Zhang F., Moon A., Childs K., Goodbourn S., Dixon L.K. (2010). The African Swine Fever Virus DP71L Protein Recruits the Protein Phosphatase 1 Catalytic Subunit To Dephosphorylate eIF2α and Inhibits CHOP Induction but Is Dispensable for These Activities during Virus Infection. J. Virol..

[6] Davis M.E., Wang M.K., Rennick L.J., Full F., Gableske S., Mesman A.W., Gringhuis S.I., Geijtenbeek T.B.H., Duprex W.P., Gack M.U. (2014). Antagonism of the phosphatase PP1 by the measles virus v protein is required for innate immune escape of MDA5. Cell Host Microbe.

[7] Richard C.A., Rincheval V., Lassoued S., Fix J., Cardone C., Esneau C., Nekhai S., Galloux M., Rameix-Welti M.A., Sizun C. (2018). RSV hijacks cellular protein phosphatase 1 to regulate M2-1 phosphorylation and viral transcription. PLoS Pathog..

[8] Li Y., Zhang C., Chen X., Yu J., Wang Y., Yang Y., Du M., Jin H., Ma Y., He B. (2011). ICP34.5 protein of herpes simplex virus facilitates the initiation of protein translation by bridging eukaryotic initiation factor 2α (eIF2α) and protein phosphatase 1. J. Biol. Chem..

[9] Michelson S., Turowski P., Picard L., Goris J., Landini M.P., Topilko A., Hemmings B., Bessia C., Garcia A., Virelizier J.L. (1996). Human cytomegalovirus carries serine/threonine protein phosphatases PP1 and a host-cell derived PP2A. J. Virol..

[10] McKinney C., Zavadil J., Bianco C., Shiflett L., Brown S., Mohr I. (2014). Global reprogramming of the cellular translational landscape facilitates cytomegalovirus replication. Cell Rep..

[11] Griffiths P., Reeves M. (2021). Pathogenesis of human cytomegalovirus in the immunocompromised host. Nat. Rev. Microbiol..

[12] Nathans R., Cao H., Sharova N., Ali A., Sharkey M., Stranska R., Stevenson M., Rana T.M. (2008). Small-molecule inhibition of HIV-1 Vif. Nat. Biotechnol..

[13] Stecher C., Marinkov S., Mayr-Harting L., Katic A., Kastner M.T., Rieder-Rommer F.J.J., Lin X., Nekhai S., Steininger C. (2021). Protein Phosphatase 1 Regulates Human Cytomegalovirus Protein Translation by Restraining AMPK Signaling. Front. Microbiol..

[14] Munday R. (2013). Is protein phosphatase inhibition responsible for the toxic effects of Okadaic acid in animals?. Toxins.

[15] Rieder F.J.J., Gröschel C., Kastner M.T., Kosulin K., Laengle J., Zadnikar R., Marculescu R., Schneider M., Lion T., Bergmann M. (2017). Human cytomegalovirus infection downregulates vitamin-D receptor in mammalian cells. J. Steroid Biochem. Mol. Biol..

[16] Rappsilber J., Mann M., Ishihama Y. (2007). Protocol for micro-purification, enrichment, pre-fractionation and storage of peptides for proteomics using StageTips. Nat. Protoc..

[17] Tyanova S., Temu T., Cox J. (2016). The MaxQuant computational platform for mass spectrometry-based shotgun proteomics. Nat. Protoc..

[18] Ritchie M.E., Phipson B., Wu D., Hu Y., Law C.W., Shi W., Smyth G.K. (2015). Limma powers differential expression analyses for RNA-sequencing and microarray studies. Nucleic Acids Res..

[19] Didusch S., Madern M., Hartl M., Baccarini M. (2022). Amica: An Interactive and User-Friendly Web-Platform for the Analysis of Proteomics Data. BMC Genom..

[20] Bailey T.L., Johnson J., Grant C.E., Noble W.S. (2015). The MEME Suite. Nucleic Acids Res..

[21] Grant C.E., Bailey T.L., Noble W.S. (2011). FIMO: Scanning for occurrences of a given motif. Bioinformatics.

[22] Larsson J., Gustafsson P. A Case Study in Fitting Area-Proportional Euler Diagrams with Ellipses Using eulerr. Proceedings of the International Workshop on Set Visualization and Reasoning.

[23] Chiang D.Y., Lebesgue N., Beavers D.L., Alsina K.M., Damen J.M.A., Voigt N., Dobrev D., Wehrens X.H.T., Scholten A. (2015). Alterations in the interactome of serine/threonine protein phosphatase type-1 in atrial fibrillation patients. J. Am. Coll. Cardiol..

[24] Chen E.Y., Tan C.M., Kou Y., Duan Q., Wang Z., Meirelles G.V., Clark N.R., Ma’ayan A. (2013). Enrichr: Interactive and collaborative HTML5 gene list enrichment analysis tool. BMC Bioinform..

[25] Hendrickx A., Beullens M., Ceulemans H., Den Abt T., Van Eynde A., Nicolaescu E., Lesage B., Bollen M. (2009). Docking Motif-Guided Mapping of the Interactome of Protein Phosphatase-1. Chem. Biol..

[26] Orchard S., Ammari M., Aranda B., Breuza L., Briganti L., Broackes-Carter F., Campbell N.H., Chavali G., Chen C., del-Toro N. (2014). The MIntAct project--IntAct as a common curation platform for 11 molecular interaction databases. Nucleic Acids Res..

[27] Bogdanow B., Gruska I., Mühlberg L., Protze J., Hohensee S., Vetter B., Bosse J.B., Lehmann M., Sadeghi M., Wiebusch L. (2023). Spatially resolved protein map of intact human cytomegalovirus virions. Nat. Microbiol..

[28] Spector D.H. (2015). Human cytomegalovirus riding the cell cycle. Med. Microbiol. Immunol..

[29] Tyl M.D., Betsinger C.N., Cristea I.M. (2022). Virus–host protein interactions as footprints of human cytomegalovirus replication. Curr. Opin. Virol..

[30] Patro A.R.K. (2019). Subversion of immune response by human cytomegalovirus. Front. Immunol..

[31] Cox J., Hein M.Y., Luber C.A., Paron I., Nagaraj N., Mann M. (2014). Accurate proteome-wide label-free quantification by delayed normalization and maximal peptide ratio extraction, termed MaxLFQ. Mol. Cell. Proteomics.

[32] Cox J., Mann M. (2008). MaxQuant enables high peptide identification rates, individualized p.p.b.-range mass accuracies and proteome-wide protein quantification. Nat. Biotechnol..

[33] Lenarcic E.M., Hale A.E., Vincent H.A., Dickmander R.J., Sanders W., Moorman N.J. (2024). Protein phosphatase 1 suppresses PKR/EIF2α signaling during human cytomegalovirus infection. J. Virol..

[34] Acharya D., Reis R., Volcic M., Liu G.Q., Wang M.K., Chia B.S., Nchioua R., Groß R., Münch J., Kirchhoff F. (2022). Actin cytoskeleton remodeling primes RIG-I-like receptor activation. Cell.

[35] Ammosova T., Jerebtsova M., Beullens M., Lesage B., Jackson A., Kashanchi F., Southerland W., Gordeuk V.R., Bollen M., Nekhai S. (2005). Nuclear targeting of protein phosphatase-1 by HIV-1 Tat protein. J. Biol. Chem..

[36] Ammosova T., Yedavalli V.R.K., Niu X., Jerebtsova M., Van Eynde A., Beullens M., Bollen M., Jeang K.T., Nekhai S. (2011). Expression of a protein phosphatase 1 inhibitor, cdNIPP1, increases CDK9 threonine 186 phosphorylation and inhibits HIV-1 transcription. J. Biol. Chem..

[37] Ammosova T., Jerebtsova M., Beullens M., Voloshin Y., Ray P.E., Kumar A., Bollen M., Nekhai S. (2003). Nuclear protein phosphatase-1 regulates HIV-1 transcription. J. Biol. Chem..

[38] Ammosova T., Platonov M., Ivanov A., Kont Y.S., Kumari N., Kehn-Hall K., Jerebtsova M., Kulkarni A.A., Üren A., Kovalskyy D. (2014). 1E7-03, a low MW compound targeting host protein phosphatase-1, inhibits HIV-1 transcription. Br. J. Pharmacol..

[39] Kim J.J., Lipatova Z., Segev N. (2016). TRAPP complexes in secretion and autophagy. Front. Cell Dev. Biol..

[40] Lučin P., Mahmutefendić H., Blagojević Zagorac G., Ilić Tomaš M. (2015). Cytomegalovirus immune evasion by perturbation of endosomal trafficking. Cell. Mol. Immunol..

[41] Mosher B.S., Kowalik T.F., Yurochko A.D. (2022). Overview of how HCMV manipulation of host cell intracellular trafficking networks can promote productive infection. Front. Virol..

[42] Close W.L., Glassbrook J.E., Gurczynski S.J., Pellett P.E. (2018). Infection-induced changes within the endocytic recycling compartment suggest a roadmap of human cytomegalovirus egress. Front. Microbiol..

[43] Hertel L., Mocarski E.S. (2004). Global Analysis of Host Cell Gene Expression Late during Cytomegalovirus Infection Reveals Extensive Dysregulation of Cell Cycle Gene Expression and Induction of Pseudomitosis Independent of US28 Function. J. Virol..

[44] Stenmark H. (2009). Rab GTPases as coordinators of vesicle traffic. Nat. Rev. Mol. Cell Biol..

[45] Krzyzaniak M.A., Mach M., Britt W.J. (2009). HCMV-encoded glycoprotein M (UL100) interacts with rab11 effector protein FIP4. Traffic.

[46] Spearman P. (2018). Viral interactions with host cell Rab GTPases. Small GTPases.

[47] Lučin P., Kareluša L., Zagorac G.B., Lučin H.M., Pavišić V., Vučko N.J., Jurić S.L., Marcelić M., Lisnić B., Jonjić S. (2018). Cytomegaloviruses exploit recycling rab proteins in the sequential establishment of the assembly compartment. Front. Cell Dev. Biol..

[48] Roberts B.S., Satpute-Krishnan P. (2023). The many hats of transmembrane emp24 domain protein TMED9 in secretory pathway homeostasis. Front. Cell Dev. Biol..

[49] Delorme-Axford E., Morosky S., Bomberger J., Stolz D.B., Jackson W.T., Coyne C.B. (2014). BPIFB3 Regulates Autophagy and Coxsackievirus B Replication through a Noncanonical Pathway Independent of the Core Initiation Machinery. mBio.

[50] Evans A.S., Lennemann N.J., Coyne C.B. (2021). BPIFB3 interacts with ARFGAP1 and TMED9 to regulate non-canonical autophagy and RNA virus infection. J. Cell Sci..

[51] Baer A., Shafagati N., Benedict A., Ammosova T., Ivanov A., Hakami R.M., Terasaki K., Makino S., Nekhai S., Kehn-Hall K. (2016). Protein Phosphatase-1 regulates Rift Valley fever virus replication. Antivir. Res..

[52] Carey B.D., Ammosova T., Pinkham C., Lin X., Zhou W., Liotta L.A., Nekhai S., Kehn-Hall K. (2018). Protein Phosphatase 1α Interacts with Venezuelan Equine Encephalitis Virus Capsid Protein and Regulates Viral Replication through Modulation of Capsid Phosphorylation. J. Virol..

[53] Ilinykh P.A., Tigabu B., Ivanov A., Ammosova T., Obukhov Y., Garron T., Kumari N., Kovalskyy D., Platonov M.O., Naumchik V.S. (2014). Role of protein phosphatase 1 in dephosphorylation of Ebola virus VP30 protein and its targeting for the inhibition of viral transcription. J. Biol. Chem..

[54] Ammosova T., Pietzsch C.A., Saygideǧer Y., Ilatovsky A., Lin X., Ivanov A., Kumari N., Jerebtsova M., Kulkarni A., Petukhov M. (2018). Protein Phosphatase 1-Targeting Small-Molecule C31 Inhibits Ebola Virus Replication. J. Infect. Dis..

[55] Lin X., Ahmad A., Ivanov A.I., Simhadri J., Wang S., Kumari N., Ammosova T., Nekhai S. (2023). HIV-1 Transcription Inhibitor 1E7-03 Decreases Nucleophosmin Phosphorylation. Mol. Cell. Proteom..

[56] Perez-Riverol Y., Csordas A., Bai J., Bernal-Llinares M., Hewapathirana S., Kundu D.J., Inuganti A., Griss J., Mayer G., Eisenacher M. (2019). The PRIDE database and related tools and resources in 2019: Improving support for quantification data. Nucleic Acids Res..

